# Epidemiological, clinical and pathological characteristics of gastric neoplasms in the province of Cremona: the experience of the first population-based specialized gastric cancer registry in Italy

**DOI:** 10.1186/s12885-019-5366-1

**Published:** 2019-03-08

**Authors:** B. M. Donida, G. Tomasello, M. Ghidini, F. Buffoli, M. Grassi, W. Liguigli, G. Maglietta, L. Pergola, M. Ratti, G. Sabadini, L. Toppo, M. Ungari, R. Passalacqua

**Affiliations:** 1 ASST of Cremona, Viale Concordia 1, 26100 Cremona, CR Italy; 2 ASST of Crema, Largo Ugo Dossena 2, 26013 Crema, CR Italy; 3 Hospital of Suzzara, Via General Cantore 14/b, 46029 Suzzara, MN Italy; 4 University Hospital of Parma, Via Gramsci 14, 43126 Parma, PR Italy; 5 University of Florence, 50121 Parma, FI Italy; 6 Istituto Figlie San Camillo of Cremona, Via Fabio Filzi 56, 26100 Cremona, CR Italy; 7 Civil Hospital of Voghera, ASST of Pavia, Via Indipendenza 34, 27100 Pavia, PV Italy

**Keywords:** Gastric cancer incidence, Population-based specialized registry, Gastric cancer survival, Gastric cancer clinical characteristics, Gastric cancer pathological characteristics

## Abstract

**Background:**

The gastric cancer incidence rate differs widely across geographical areas. In Italy, in the province of Cremona the incidence is high, compared to the national situation. For this reason a specialized population-based registry was set up.

**Methods:**

The collection encompasses all gastric cancers diagnosed in the three districts of the province since January 1, 2010. The main data sources were the pathological and Hospital Discharge Records and patient clinical charts. Only diagnoses of primary gastric cancer were considered. For each case the following variables were registered: personal data, medical history and symptoms at diagnosis; imaging assessments performed, details on surgery and other treatments received; genetic background and biomolecular characteristics; social and environmental factors.

**Results:**

As of November 2017, 1087 cases were collected; of which 876, diagnosed up to December 2015, were analyzed. Male/female ratio was 1.4. The European Age-standardized Incidence Rate was 41.4 for males and 28.3 for females as compared to a national average of 33.3 and 17.0 respectively. Median age at diagnosis was 73 for male and 78 for female. Helicobacter Pylori infection was present in fewer than 20% of cases. HER-2 gene was amplified in about 25% of cases. Primary tumour location was the gastro-esophageal junction or cardia in 17.5% in males and 8.3% in females. The majority of cases (58.3%) were diagnosed at an advanced stage and overall only 41.2% underwent surgery. Median overall survival was 14.8 months for men and 18.5 for women. Age standardized 5-year relative survival was 31.4% for men and 40.5% for females. Neoadjuvant treatment was performed in fewer than 10% of patients who underwent surgery, and the rate of postoperative therapy adherence was low.

**Discussion:**

This study shows a high gastric cancer incidence in the province of Cremona, with a geographical spread across different districts. Moreover, a high percentage of gastric cancers were detected at an advanced stage of disease and a low rate of 5-year relative survival was registered. Based on these findings, effective preventive interventional health strategies and screening procedures need to be implemented to reduce the impact of this pathology in this geographical area.

## Background

Despite its declining incidence, gastric cancer (GC) is still the fifth most common malignancy in the world and remains the third leading cause of cancer-related death in both sexes worldwide, following lung and liver cancer [1]. Except for Japan [2], where screening programs have already been implemented, the 5-year survival rate for patients (pts) with GC is generally poor. In western populations, including Europe and the United States, 5-year survival rates usually do not exceed 25–30% [3, 4]. Epidemiology of GC is characterized by a wide variation in incidence and mortality rates across different geographical areas, as reported in GLOBOCAN [1]. Most GC cases originate in Eastern Asia (58.1%), Europe (14.7%) and parts of Central and Latin America (7.8%) [1]. Differences in epidemiology have prompted and guided cancer research and cancer control strategies. For example, in Japan, due to the high incidence, active surveillance and preventive gastric resection are performed [5, 6]. In Italy, according to the latest published data [7], two geographical areas are mainly associated with high incidence and mortality rates: one in northern Italy and the other one in the middle of the country. In particular, in the northern part, the province of Cremona presents high GC mortality rates. This has been noticed since 1996 [8] but the province lacks accurate data to monitor the situation. To investigate more deeply the real epidemiological situation for inhabitants of the province of Cremona, a specialized population-based GC registry was set up at the Oncology Department of the public Hospital “ASST” of Cremona. This article aims to report the results of the first six years of such data collection. Cancer registries have a key role in cancer control. Owing to the very poor prognosis of stomach cancer, the results of epidemiological and experimental studies are fundamental to develop primary prevention strategies [9].

## Methods

The project is an observational and descriptive epidemiological study, complemented by a retrospective chart review. Official collaboration with intra and extra-provincial facilities was established, in order to have access to pt data, provided that they were formally resident in the province of Cremona. A multidisciplinary team of clinicians and pathologists was involved in the project. All medical reports with diagnosis of GC were reviewed. For each incident case the following variables were registered: medical history and information about diagnosis, imaging assessments performed, details of surgery and other treatments received, genetic background and biomolecular characteristics, social and environmental factors. All data were collected, recorded, protected and processed in accordance with the cancer registration recommendations given by AIRTum (Associazione Italiana Registri Tumori) and IARC (International Agency for Research on Cancer) and in accordance with the national privacy laws [10–12].

### The territory

**Fig. 1:**
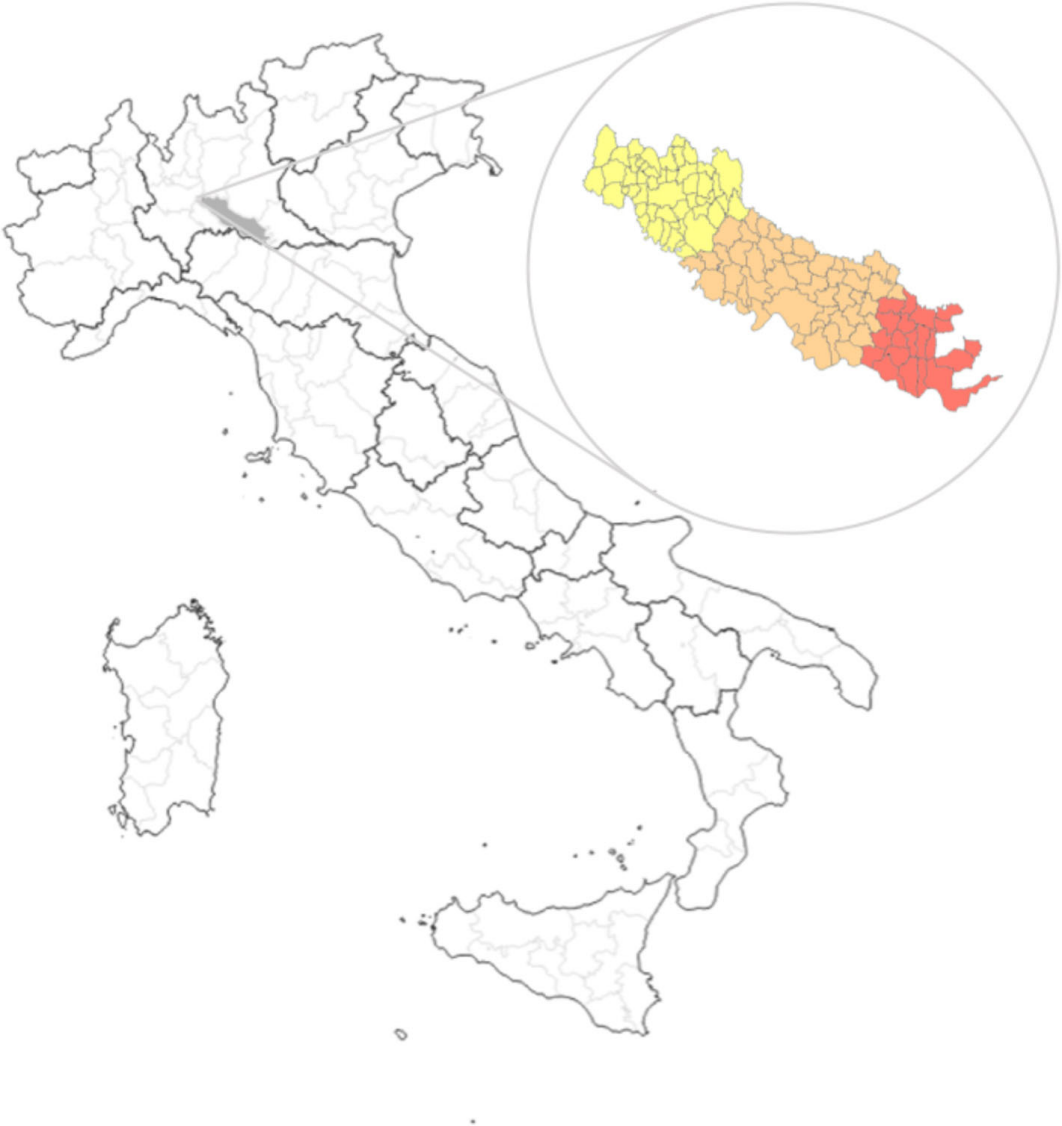
The territory of the province of Cremona. The province of Cremona is part of the Po River Plain belonging to the Lombardy region, in the north of Italy. The total area of the province of Cremona, filled in light grey, is about 700 mile^2^ with a total of 115 municipalities. As reported in the box on the right, it is divided into three districts: Crema (48 municipalities, yellow fill colour), Cremona (47 municipalities, orange fill colour) and Casalmaggiore (20 municipalities, in red colour). On the map of Italy, the bold border corresponds to the area of a region and the light border to the area of provinces included in each region. On the map of the province of Cremona, the borders delineate the municipalities of the province. Image produced by QGIS 2.8.3-Wien and GIMP 2.10.4 software; data available at https://www.istat.it/it/ (latest access on July 19, 2018)

The province of Cremona is in northern Italy and covers an area of 1770.46 km^2^. The territory is divided into three districts: Crema (the northern), Cremona (the central) and Casalmaggiore (the southern district), with a total of 115 municipalities. “Fig. 1”.

### The population

According to the 2017 (December 31, 2016) census, the total population of the province of Cremona is 359,388.00 inhabitants. The male (M)/female (F) ratio is 1.03 and the mean age 44.7 years. Race is predominantly Caucasian.

### Cases and variables collected

Any age at diagnosis was included. At time of diagnosis pts had to be inhabitants of the province of Cremona. Otherwise as cancer registration guidelines recommended, “in situ” tumours (Tis) were also included because the purpose of this study is to give an overall summary of the incidence of the gastric neoplasm in the province of Cremona. For the same reason, we did not record cases diagnosed based on Death Certificate Only (DCO) without additional clinical data available. Overall, Tis collected and DCO excluded concerned a very limited number of cases. Only diagnoses of primary gastric neoplasms were considered. Precancerous lesions and relapsed tumour were not considered. Primary tumour location was stomach or gastro-esophageal junction (GEJ), according to the Union for International Cancer Control (UICC), 7th edition [13, 14]. Location was codified as distal esophagus (only adenocarcinoma, ADK), GEJ, cardia, fundus, body, antrum, pylorus and lesser or greater curvature of the stomach. For the analyses, tumour location was divided into three subgroups (GEJ-cardia, fundus-body, including lesser and greater curvature, and antrum-pylorus). All different histologies were considered: gastric cancer, lymphoma, sarcoma and Gastro-Intestinal Stromal Tumour (GIST). Gastric cancer was classified according to the Lauren classification system, which distinguishes two main histological types: “intestinal” and “diffuse” (DGC) [15]. “Mixed” gastric carcinomas, composed of intestinal and diffuse components, have also been identified [16, 17]. The TNM classification was recorded and the corresponding pathological stage was determined according to the 7th edition of the UICC [13, 14]. Evaluation of Helicobacter pylori (HP) infection was performed by immunohistochemistry (IHC) in healthy gastric mucosa using the GIEMSA stain method. HER-2 oncogene over-expression was evaluated in tumour gastric mucosa by IHC method Dako Hercep Test™ (R&D Systems, Minneapolis, MN, USA). Results were confirmed by Fluorescent in Situ Hybridization (FISH) when IHC positivity score was two or more [18].

### Hereditary diffuse gastric Cancer

To identify Hereditary Diffuse Gastric Cancer (HDGC) cases, the updated guidelines of the International GC Linkage Consortium (IGCLC) that included revised CDH-1 testing criteria were used [19–21].

### Statistical analysis

Epidemiological indicators were calculated according to IARC guidelines [12]. To compare our findings to other Italian areas, Age Standardized Incidence Rates (ASIRs) were calculated using the new standard European population (EU-ASIR), defined by Eurostat, the statistical office of the European Union, in 2013 and introduced for the first time in the latest AIOM-AIRTum publication “I numeri del cancro 2017” [7, 22–24]. To compare incidence to worldwide areas, we used the age-structure of the world standard population (W-ASIR) [25–27]. The interval of confidence (CI) of ASIR was calculated using the approximation to Poisson distribution. The trend of incidence, expressed as Annual Percent Change (APC) was evaluated by Join Point v. 4.5.0.1 (National Cancer Institute, Bethesda, MD) [28, 29]. To investigate whether a difference in incidence was present across the area, the ASIR of each municipality was calculated, grouped by district of belonging and tested by the non-parametric Kruskal-Wallis test. According to these preliminary results, a spatial analysis has to be planned and will be performed. The age-standardized 5-year relative survival was evaluated using a period approach [30–33]. Overall survival (OS) and progression free survival (PFS) analyses were carried out by Kaplan–Meier (K-M) methods [34] and significant differences evaluated by Log-Rank Test or by Wilcoxon-Breslow-Gehan test, when required [35]. In PFS analysis, if no progressive disease (PD) was confirmed by RECIST criteria or death occurred, data were censured at the time of the last follow up (FU). The simultaneous effect of several variables on OS was investigated using the semi-parametric Cox proportional hazards regression model. The backwards stepwise method was used to define the last model. Descriptive statistics were used to summarize data and the Chi Square Test (X^2^) or the Analysis of Variance (ANOVA) were used to compare groups. Statistical analysis was carried out by STATA 13 (Texas, USA). A *p*-value below 0.05 was considered as statistically significant.

## Results

### The data collection

As of November 2017, 1087 cases were collected and 876 cases diagnosed up to December 2015 were analysed. Histological confirmation was available for 92.7% of cases, while the remainder were GC cases diagnosed clinically and by radiological assessment as computed tomography scan, CT scan, or abdominal ultrasound without tissue biopsy (data obtained from Hospital Discharge Records, DCO and medical charts). Five hundred and thirteen cases (58.6%) were M and 363 (41.4%) F, with a sex ratio of 1.4.

### Symptoms leading to GC diagnosis

Data was investigated in 538 GC pts. Symptoms recorded were, in decreasing order of frequency: anaemia (41.5%), weight loss (30.4%), epigastralgia (24.3%), dyspepsia (19.0%), melena (15%), dysphagia (12.5%), fatigue (11.7%), vomiting (10.8%), hematemesis (8.2%), loss of appetite (5.8%), heartburn (4.6%), physical decline (3.0%), nausea (2.8%), bowel obstruction (0.9%) and itching (0.4%). Differences were seen between gender for anaemia (*p* = 0.002) and vomiting (*p* = 0.020), more frequent in F; and for dysphagia (p = 0.002), more frequent in M. According to stage of disease, anaemia (*p* = 0.006) and hematemesis (*p* = 0.038) seemed to be associated with early diagnosis; weight loss (*p* = 0.023), dysphagia (p = 0.020) and lack of appetite (p = 0.006) with diagnosis in advanced stage of disease. Among 314 pts, a history of gastritis was recorded in 38.9% of pts, a previous HP infection in 22.5%, peptic ulcer in 20.5%, acid reflux disease in 12.2% and a previous gastro-resection for ulcer in 11.7% of pts. No differences by gender were detected.

### The epidemiological characteristics

In the province of Cremona, median age (IQR; range min-max) at diagnosis was 73 (57–89; 30–94) for M; and 78 (64–92; 37–100) for F. Difference between sexes was statistically significant (*p* < 0.001) and no variation was registered over years (*p* = 0.746 in M and *p* = 0.488 in F). EU-ASIR (CI 95%) was 41.39 (35.35–48.47) for M and 28.30 (24.17–33.14) for F. Similarly, the W-ASIR (CI 95%) was 20.76 (19.20–22.31) for M and 12.74 (11.21–14.27) for F, while Italian averages are 10.9 and 5.6 respectively [1]. Incidence was decreasing over years, with an APC by − 1.92% for men and − 3.21% for women (slopes not significant). There seemed to be a difference in incidence across districts, both in M (*p* = 0.004, d.f. 2) and in F (*p* = 0.003, d.f. 2) with fewer cases detected in the northern district. Age standardized 5-years relative survival (CI 95%) was 31.44 (26.41–36.69) in M and 40.50 (34.21–46.88) in F. “Table 1”.

**Table 1:** Epidemiological characteristics of GC: the province of Cremona and the national data

Epidemiological characteristic of GC	Male	Female
Median age at diagnosis (IQR; min-max)		
Province of Cremona	73 (57–89; 30–94)	78 (64–92; 37–100)
European Age Standardized Incidence Rate ASIR × 100,000 (IC 95%)		
Province of Cremona	41.39 (35.35–48.47)	28.30 (24.17–33.14)
Crema	34.20 (29.20–40.05)	21.40 (18.28–25.06)
Cremona	48.15 (41.12–56.39)	32.35 (27.62–37.88)
Casalmaggiore	39.97 (34.14–46.81)	34.77 (29.70–40.72)
Italy [7]		
Italy, north	35.9	17.7
Italy, centre	39.3	20.5
Italy, south and islands	24.8	12.8
Annual Percent Change APC (IC 95%)		
Province of Cremona	− 1.92 (− 7.6;+ 4.1)	− 3.2 (− 6.5;+ 0.2)
Italy [7]	− 3.4	− 3.0
No. of people to be followed for a new diagnosis of GC		
“0–49” year age class		
Province of Cremona	548	1460
Italy [7]	1070	1235
“50–69” year age class		
Province of Cremona	72	147
Italy [7]	122	250
“70–84” year age class		
Province of Cremona	30	41
Italy [7]	45	94
“During all life” (0–84)		
Province of Cremona	21	32
Italy [7]	32	65
Age standardized 5-years relative survival (%, IC 95%)		
Province of Cremona	31.44 (26.41–36.69)	40.50 (34.21–46.88)
Italy [7]		
Italy, north west	31	36
Italy, north east	33	34
Italy, centre	32	38
Italy, south and islands	28	27
1-year risk of death (since diagnosis, %)		
Province of Cremona	31.36	28.86

The national data were from the latest published official data [7], that reported the pull of incidence 2008–2013

### Hereditary cases

Seven out of 876 pts were diagnosed with GC before the age of forty and two of them (0.23%) were DGC, according to II IGCLC criteria (*“Individuals with DGC before the age of 40”,* [19–21]). Hereditary anamnesis to investigate I and III IGCLC criteria, was available for 242 pts. One out of 242 fulfilled the I criteria (“*Families with two or more pts with GC at any age, one confirmed DGC”,* [19–21]). One out of 242 fulfilled the III criteria (“*Families with both DGC and Lobular Breast Cancer (LBC), one diagnosis before the age of 50”* [19–21]).

Besides IGCLC criteria and pts that may be HDGC, the investigation of hereditary anamnesis showed that in about 15 families of pts with intestinal ADK diagnosed after 50, three to five relatives (I and II degree) had diagnosis of GC.

### Histopathological and biomolecular characteristics

In our analyses an association between sex and cancer site (*p* < 0.001, 2 d.f.) was found, with M having more proximal cancer. The principal histotype recorded was ADK with more than 90% of GCs registered in both sexes. An association between ADK histotype and sex was recorded (*p* = 0.002, 1d.f.), with a predominance of DGC in F. The presence of HP was investigated in 567 cases: 19.9% of M and 15.7% of F proved positive. While no association with sex was detected, an association between presence of HP and district of residence was seen (*p* < 0.001, 2 d.f.). In detail, percentages were 12.8 for the district of Crema, 26.0 for Cremona and 16.1 for Casalmaggiore. HER-2 gene resulted amplified more in M (29.6%) than in F (21.4%) with difference by gender statistically significant in resected GCs (*p* = 0.027, 1 d.f.). No difference according to the district of residence was evident. Overall, and without differences by gender, 41.7% of pts were diagnosed at initial stage (TNM stage I and II) and the remaining 58.3% at an advanced stage of disease (TNM stage III or IV). Data were summarized, grouped by sex and by status of surgery, in “Table 2”.

**Table 2:** GC in the province of Cremona: pathological and clinical characteristics of incident cases (all cases and grouped by surgery) total and by sex

Factor	Total incident cases			Resected GCs			Not-resected GCs		
	Total (N)	M (%)	F (%)	Total (N)	M (%)	F (%)	Total (N)	M (%)	F (%)
Number of cases	876	58.6	41.4	361	40.5	42.1	515	59.2	40.8
Primary tumour location									
Cardias-GEJ	106	17.5	8.3	32	12.9	4.1	74	21.2	11.9
Body-Fundus	366	50.2	42.9	164	49.5	43.5	202	50.8	42.4
Antrum-Pylorus	304	32.3	48.8	153	37.6	52.4	151	28.0	45.8
Not defined^§^	100			12			88		
Tumour Histotype									
Intestinal ADK	538	68.0	58.5	217	66.3	51.6	321	69.2	64.0
Diffuse /mixed ADK	243	24.9	34.8	127	28.9	43.8	116	22.2	27.5
Others	58	7.1	6.7	17	4.8	4.6	41	8.6	8.5
Tumour Grading (ADKs)									
G1	29	5.1	7.6	19	5.2	6.5	11	4.4	7.3
G2	204	46.2	35.7	151	51.5	37.0	68	36.3	32.9
G3–4	103	48.7	56.7	162	43.3	56.5	116	59.3	59.8
HP infection (presence of HP/evaluated cases)									
Province of Cremona	103/567	19.9	15.7	49/256	20.0	18.0	54/311	19.8	13.7
Crema	24/228	12.8	7.4	9/95	16.7	21.1	15/133	32.4	17.6
Cremona	69/275	26.0	23.5	37/130	76.7	73.7	32/145	59.5	58.8
Casalmaggiore	10/64	16.1	15.2	3/31	6.7	5.3	7/33	8.1	23.5
HER-2 status (amplified/evaluated cases)									
Province of Cremona	55/205	29.6	21.4	30/97	38.7	17.1	25/108	21.9	25.7
TNM Stage at diagnosis									
Stage I	122	20.2	27.6	82	20.0	27.7	40	20.5	27.4
Stage II	98	17.7	20.0	97	26.8	28.4	1	0.9	0
Stage III	131	25.9	23.3	130	39.5	33.1	1	0.9	0
Stage IV	176	36.3	29.0	44	13.7	10.8	132	77.7	72.6
Not available^§^	349			8			341*		

Legend: *M* male, *F* female, *ADK* Adenocarcinoma, *G* Tumour grading, # numbers and not percentages were reported for these values, § data not included in the statistical analyses, * pts who did not have surgery (no pathological staging) for whom clinical staging was unavailable

### Surgery

Data on surgery was available for 354 M (69.0% of total cases) and 253 F (69.7% of total cases). Forty point five percent of M and 42.1% of F respectively underwent surgery. Overall 50.7% of resected GCs were diagnosed at early stage (TNM I-II) and the remaining 49.3% in advanced stage (TNM III-IV), with no differences by gender. In contrast, between pts who did not have surgery, 23.0% had an endoscopy resolution (T*is*, No. = 34 and T1 cases, No. = 6), 70.0% did not have surgery because they were diagnosed at an advanced metastatic disease and the remaining 7% (17 pts overall, of which 2/17 with a defined TNM staging) were not metastatic, but did not have surgery because of co-morbidity. “Table 2” summarizes the data. The logistic regression model showed a significantly decreased probability (− 54.2%) to undergo surgery with increasing stage at diagnosis (HR =0.458; OR IC 95%: 0.378–0.555, *p* < 0.001) while the age at diagnosis did not seem to affect this probability. Overall, 38.8% of surgeries performed a D1 lymphadenectomy, while in the remainder a D2–3 lymphadenectomy was made. Over the years, if no difference was detected in the D1/D2–3 lymphoadenectomy ratio (*p* = 0.093, 5 d.f.), a significant difference was detected in the number of lymph nodes (LNs) collected (*p* = 0.001, 5 d.f., Bonferroni’s correction), with the mean increasing from 24.28 (Standard Deviation, SD 13.38) in 2010 to 29.75 (SD 14.85) in 2015. No difference was detected in the number of positive lymph nodes depending on the year of surgery (*p* = 0.889, 5 d.f.; mean number equal to 5.23 ± 7.55) or by gender (5.78 ± 7.68 in M and 4.47 ± 7.31 in F; *p* = 0.118, 1 d.f.).

### Therapeutic approaches

Pts who received therapy before surgery were 7.6% of resected GCs. No statistically significant difference was detected by gender (*p* = 0.065, 1 d.f.) or across years (*p* = 0.232, 5 d.f.), even if an initial reversion of trend seemed to occur. In detail, percentages of pts that received neoadjuvant therapies (neoadj), were 7.0 in 2010; 9.3 in 2011; 7.1 in 2012; 4.2 in 2013; 3.9 in 2014 and 15.4% in the year of diagnosis 2015. Forty-one percent registered a postoperative therapy adherence, proceeding with the treatment after surgery. Data on adjuvant therapy (adj) was available for 176/353 resected GCs: 16.77% received the treatment. No statistically significant differences were detected by gender (*p* = 0.929, 1 d.f.) or across years (*p* = 0.708, 5 d.f.). Type of adj was chemotherapy (CT) in 42.59% of cases, a combination of CT and radiotherapy (RT) in 50% and experimental treatment (clinical trial enrolment) in the remaining 7.41%. CT was monotherapy in 30.4% of cases.

In pts who did not have surgery and for whom data on therapies were available, a first line CT was made in 77% of cases. A second line was performed in 48.05% (37% of total), a 3rd line in 32.43% (12% of total) and of these, 5.41% (2% of total) received also a 4th line of therapy.

### Survival analysis

**Fig. 2:**
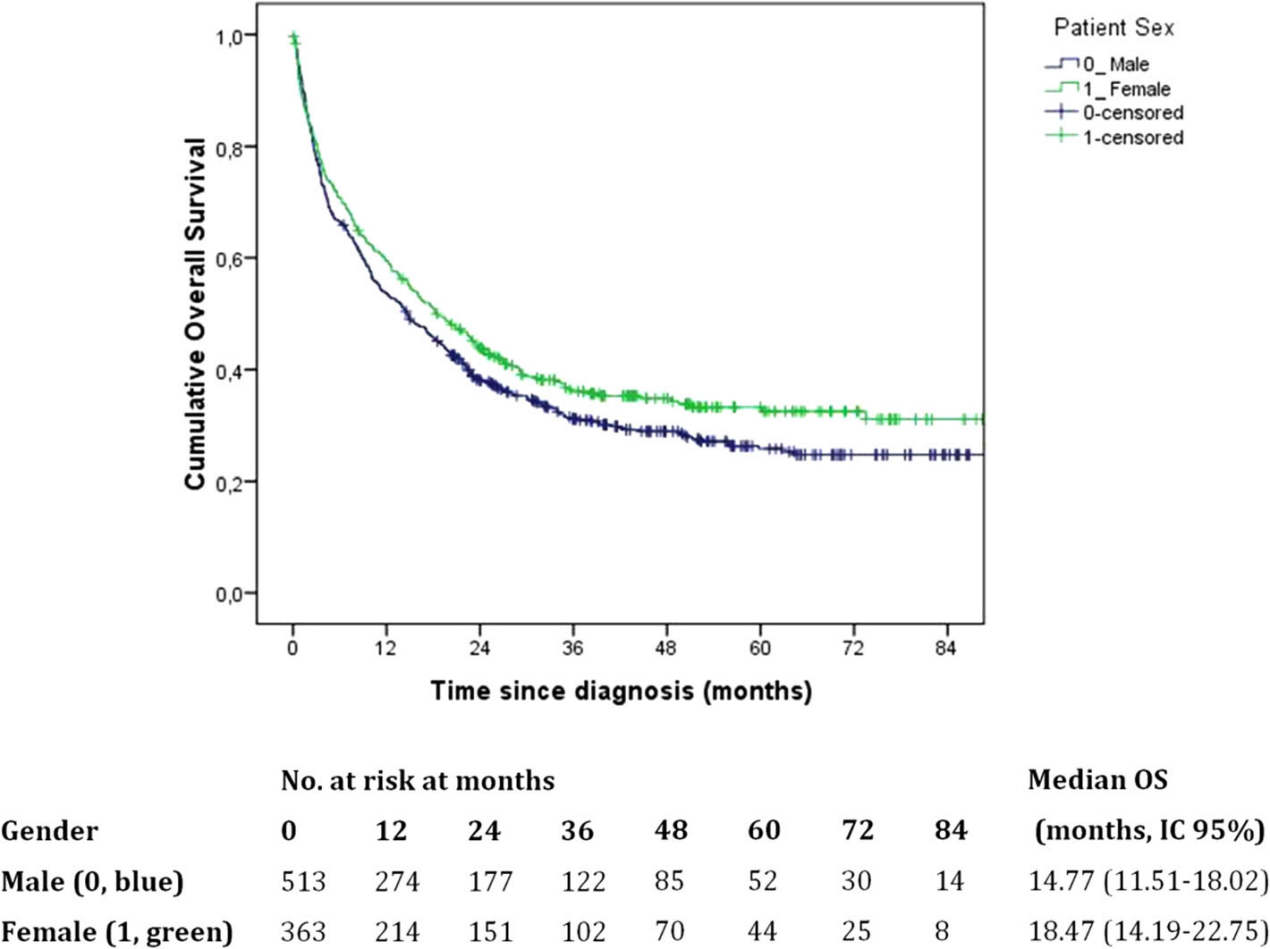
Overall survival in all GC incident cases in the province of Cremona: Kaplan-Meier curves by sex. Blue line: male, green line: female. Difference by gender was not statistically significant

**Fig. 3:**
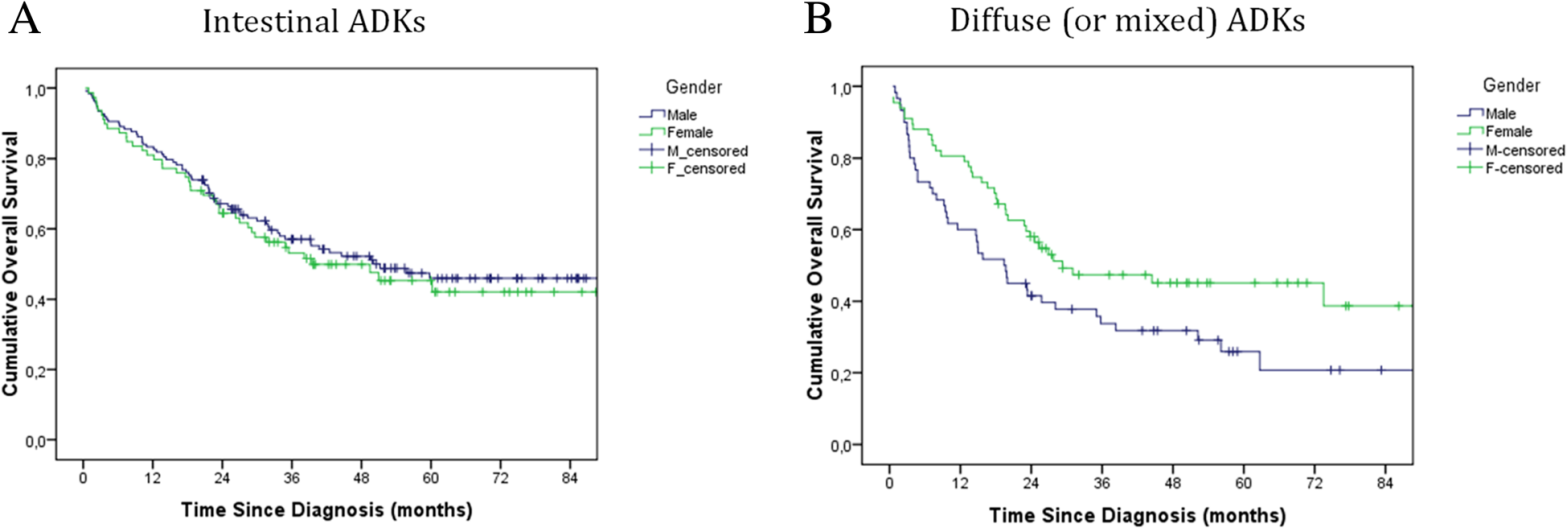
Overall survival in resected GC incident cases in the province of Cremona: Kaplan-Meier curves by sex. Panel **a**: Intestinal ADKs. Panel **b**: Diffuse/mixed ADKs. In both panels, blue line: male, green line: female

A final FU was made in November 2017 and data were available for 99.54% of cases (4/876 were considered “Lost to FU” because of unavailability at registry offices). Median FU time was 15.7 months (mm) (IQR 3.66–36.73) and overall, 68% of M and 62% of F were dead. Median OS since diagnosis was 16.27 mm (CI 95% 13.77–18.76), 14.77 mm (CI 95% 11.51–18.02) for M and 18.47 (IC95% 14.19–22.75) for F (no significant difference). K-M curves were reported in “Fig. 2”. Analyzing OS by subgroups of tumour histology, pts with a diffuse/mixed ADK performed worse than intestinal ones (*p* = 0.005, 1 d.f.). No difference in OS was detected by gender in intestinal ADKs (HR = 1.108 (CI95% 0.751–1.635; *p* = 0.604). On the contrary, for diffuse/mixed ADKs, F pts were better than M (HR = 0.617 (IC95% 0.396–0.962; *p* = 0.033), and this was verified only for pts who did not have adj-CT, while no difference by gender was detected for pts who did adj-CT. In “Fig. 3” the K-M curves are reported.

The number of positive LNs increased the risk of death by 3.4% for each additional positive LN detected during surgery, for equal gender and disease stage, with a statistically significant result (HR = 1.034, CI 95% 1.015–1.054, *p* < 0.001).

As expected, statistically significant differences in median OS were recorded, in M as well as in F, considering the TNM staging at diagnosis (*p* < 0.001, 3 d.f.) while no differences were recorded analyzing data comparing M and F in each stage of disease.

PD was confirmed in 49.23% of M and in 40.58% of F pts, where data was available (130 M and 69 F). No difference in number of pts who underwent PD was registered between sexes (*p* = 0.48, 1 d.f.). Median time to PD was 27.43 mm (IC 95% 3.79–51.08) with no significant differences by gender (*p* = 0.242, 1 d.f.). The median OS after PD confirmation decreased to 7.87 mm (CI95% 5.78–9.95), with no differences recorded between sexes (*p* = 0.524, 1 d.f.).

Factors included in the multivariable model were: sex, age at diagnosis (</≥ 65 years), tumour location (GEJ-cardia vs non-cardia tumour), tumour histology (intestinal vs mixed/diffuse ADK), grade of differentiation of tumour cells (G1–2 vs G3–4), type of surgery (partial vs total), TNM staging (each category was compared to the previous one), resection border (negative vs positive), type of lymphadenectomy (D1 vs D2–3) and the performing of adj CT (no vs yes). The final model is reported in “Table 3”.

**Table 3:** Resected GC incident cases in the province of Cremona : prognostic factors on OS

Prognostic Factor	HR (CI 95%)	*p*-value
Female (vs male)	0.613 (0.376–0.999)	0.049*
Diffuse ADK (vs intestinal ADKs)	2.188 (1.353–3.538)	0.001*
TNM Staging		< 0.001*
TNM Staging II	0.408 (0.158–1.056)	0.065
TNM Staging III	0.436 (0.227–0.840)	0.013*
TNM Staging IV	0.811 (0.479–1.373)	0.435
Have adj-CT treatment (vs haven’t)	0.294 (0.164–0.524)	< 0.001*

*was for a significant difference at *p*-value 0.05. For the TNM staging each category was compared to the previous one

## Discussion

This work represents the first specialized GC registry in Italy. In the province of Cremona, diagnosis of stomach cancer is more frequent in M (1.4 M/F ratio) and in the elderly population (75 years old is the overall median age at diagnosis), as reported in literature [7]. In addition, in this geographical area, M are diagnosed at an earlier age than F (73 vs 78, *p* < 0.001). With about 360,000 people and about 150 cases per year, the AISRs (per 100,000 inhabitants) are 41.4 for M and 28.3 for F. These values clearly deviate from other Italian areas (35.9 in northern Italy, 39.3 in central Italy and 24.8 in southern Italy for M and 17.7 in northern Italy, 20.5 in central Italy and 12.8 in southern Italy for F [7]). More than one M out of 20 and one F out of 30 living in this area are at risk of developing GC during their lifetime. In the province of Cremona the GC incidence has declined by − 1.92% in M and − 3.21% in F respectively, even if a longer period of observation is needed to confirm this with a statistically significant interval of confidence. Comparing the rates of incidence to the world standard population, ASIRs amount to 20.8 for M and 12.7 for F, despite Italian average rates of 10.9 and 5.9, respectively [1]. The F incidence rate found in our registry is much closer to the rate reported by IARC (and amounting to 13.8) for the same gender of the Eastern Asian population, known to be a country characterized by a higher GC incidence [1].

Moreover there seems to be a lower incidence in the northern district of the province, with a statistically significant result, but more appropriate spatial analysis is needed to better investigate whether a geographical spread of incidence did exist across this area. Registry data show a significant association between sex and primary cancer location (*p* < 0.001), with a prevalence of proximal cancer (including GEJ) in M, as well as a significant association between sex and ADK histotype (*p* < 0.05), with a prevalence of DGC in F. HER-2 gene amplification was detected in 26% of cases (data consistent with literature). According to available evidence from literature, the HP is present in more than 90% of non-cardia GC [36]. The percentage of HP infection in the inhabitants of the province of Cremona, equal to 18%, is inconsistent with this known data but it should be considered that these percentages cannot be directly compared each other because 18% is the percentage based on the confirmation of the presence of HP in the histological report and moreover includes all GC tumours. HP infection has been shown to be significantly associated with district of residence (*p* < 0.001), with a lower presence of bacteria in pts living in the northern district. However, the rate still remains far below the percentage of infection usually reported in literature and this needs more accurate investigations. Seven point six percent of resected GCs received neoadj treatment and only 40% had postoperative CT. Sixteen point eight percent of resected GCs received adj therapies and overall their OS was improved (*p* < 0.001), regardless of sex or tumour histotype. Overall, 58.3% of pts were diagnosed in advanced stage (III-IV) and 41.3% underwent surgery. Median OS was 16.3 mm (95% CI, 13.8–18.8) and age standardized 5-years survival was 31.44% for M and 40.50% for F. In 49.2% of cases, pts developed PD in a mean time of 24.13 mm since diagnosis. For these pts, median OS decreased to 7.87 mm (CI95% 5.78–9.95) after PD confirmation. As expected, a strong association was detected in OS by disease stage (p < 0.001) in M as well as in F. In resected DGC, F pts seemed to fare better compared to M. This consideration is clearly hypothesis-generating and needs further investigation, taking into account several possible factors including different hormonal settings, for example. Multivariable regression analysis in resected GCs, showed type of ADK (HR_DGC vs intestinal ADK_ = 2.19, *p* = 0.001) stage of disease (*p* < 0.001, 3 d.f.) and adj-CT (HR_yes vs no_ = 0.29; *p* < 0.001) as prognostic factors on OS.

The majority of GCs are sporadic, but 1–3% are a form of HDGC [37]. In small group of South Korean, Japanese and Portuguese people, pathogenic germline CDH-1 mutations in HDGC and early-onset GC ranged between 8 and 15% [38–41]. Previous studies reported that the frequency of CDH-1 mutation is inversely proportional to the rate of incidence [42, 43]. Analyzing data according to IGCLC criteria [19–21], we can assume that hereditary predisposition does not seem one of the main reasons for high GC incidence in the province of Cremona, but it is still premature to draw definitive conclusions. At the same time, the low rate of HP infection warrants a deeper analysis by considering the ten years before diagnosis [44] and the investigation of the real prevalence of HP infection in the inhabitants of this area. The latest IARC Working Group did not consider GC as a public health priority, despite its high morbidity and mortality, and very few countries have made efforts to control its incidence up to now [45]. The only exception is the Republic of Korea, with an established nationwide screening program performed by CT scan every two years in people over 40 years of age [46]. In Japan in 2013, the government established a national health program of antibiotic treatment against HP in pts with chronic gastritis confirmed by endoscopy [47]. In Taiwan and China, during screening for colorectal cancer the evaluation of HP infection is included. Subjects with infection are included in a free endoscopy screening and free antibiotic treatment [48]. In Latin America, Chile has set up a screening program to detect HP infection by endoscopy and preventive treatment in symptomatic adults over 40 years of age [49]. Beside these countries, in the rest of the world few public health programs have been set up in order to prevent GC [50, 51]. However, it is advisable that all countries characterized by a high GC incidence should also include GC in their national cancer control plans, in order to lessen the human and economic impact of this cancer. Other plausible factors to take into account for such a high incidence rate can be found in local dietary factors or environmental causes, such as contaminated water and pesticides. The area of Cremona is characterized by a plain surrounded by rivers. Literature suggests that one important source of exposure to potential carcinogens in agro ecosystems is through water contamination by agrichemicals [52]. Agriculture represents the principal economic resource of the area, which mainly produces corn [53].

The high rate of GC incidence and differences across districts, low rate of HP infection and high percentage of pts diagnosed at an advanced stage highlight the importance of early cancer diagnosis and deeper investigation of causes in the province of Cremona.

## Conclusion

Our findings confirm and support the IARC position, which suggests and advises a deep investigation into the primary causes of GC in order to improve preventive interventional health strategies and screening procedures. High GC incidence regions, such as the province of Cremona, should consider the development of screening programs in order to increase the rate of early diagnosis and to prolong survival.

## Abbreviations

adj: Adjuvant therapy
ADK: Adenocarcinoma
AIRTum: Associazione Italiana Registri Tumori
APC: Annual Percent Change
ASIR: Age Standardized Incidence Rate
CDH-1: E-cadherin type 1
CI: Interval of Confidence
CT scan: Computed Tomography scan
CT: Chemotherapy
d.f.: Degrees of freedom
DCO: Death Certificate Only
DGC: Diffuse Gastric Cancer
EU: European
F: Female
FISH: Fluorescent in Situ Hybridization
FU: Follow Up
G: Grading
GC: Gastric Cancer
GEJ: Gastro–Esophageal Junction
GIST: Gastro-Intestinal Stromal Tumour
HDGC: Hereditary Diffuse Gastric Cancer
HER-2: Human Epidermal growth factor Receptor 2
HP: Helicobacter pylori
HR: Hazard Ratio
IARC: International Agency for Research on Cancer
IGCLC: International Gastric Cancer Linkage Consortium
IHC: ImmunoHistoChemistry
IQR: Interquartile Range
K-M: Kaplan–Meier
LN: Lymph Nodes
M: Male
mm: Months
neoadj: Neoadjuvant therapy
No: Number
OS: Overall survival
p: *p*-value
PD: Progressive Disease
PFS: Progression Free Survival
pt: Patient
RECIST: Response Evaluation Criteria In Solid Tumors
RT: Radio-Chemotherapy
SD: Standard Deviation
Tis: In situ Tumour
TNM: Tumor-Nodes-Metastasis classification
UICC: Union for International Cancer Control
vs: Versus
W: World
